# The double burden of human resource and HIV crises: a case study of Malawi

**DOI:** 10.1186/1478-4491-6-16

**Published:** 2008-08-12

**Authors:** David McCoy, Barbara McPake, Victor Mwapasa

**Affiliations:** 1 Centre for International Health and Development, University College London, 30 Guilford Street, London, WC1N 1EH, UK; 2 Institute for International Health and Development, Queen Margaret University, Edinburgh, EH12 8TS, UK; 3 Division of Community Health, College of Medicine, University of Malawi, Blantyre, Malawi

## Abstract

Two crises dominate the health sectors of sub-Saharan African countries: those of human resources and of HIV. Nevertheless, there is considerable variation in the extent to which these two phenomena affect sub-Saharan countries, with a few facing extreme levels of both: Lesotho, Zimbabwe, Zambia, Mozambique, the Central African Republic and Malawi.

This paper reviews the continent-wide situation with respect to this double burden before considering the case of Malawi in more detail. In Malawi, there has been significant concurrent investment in both an Emergency Human Resource Programme and an antiretroviral therapy programme which was treating 60,000 people by the end of 2006. Both areas of synergy and conflict have arisen, as the two programmes have been implemented. These highlight important issues for programme planners and managers to address and emphasize that planning for the scale-up of antiretroviral therapy while simultaneously strengthening health systems and the human resource situation requires prioritization among compelling cases for support, and time (not just resources).

## Background

Two crises dominate the health sectors of sub-Saharan countries: those of human resources and of HIV. In principle, both these crises magnify each other. HIV places a significant additional load on the health workforce and contributes to attrition from it through illness, caring for family members who have developed AIDS and death. And the impact of the HIV crisis is accentuated because health workers are unavailable to implement anti-HIV interventions.

A particular source of recent concern has been the impact on workforce distribution of increased levels of support for HIV/AIDS programmes and especially treatment. This paper seeks to explore this interaction in more detail. It reviews the continent-wide distribution of the two phenomena and initial evidence of the impact of expanded treatment programmes, before looking in depth at the case of Malawi, a country with one of the lowest densities of human resources for health and one of the highest prevalence rates of HIV.

## Methods

This paper is based on data derived from published literature; the global atlas of the health workforce, a database compiled by the World Health Organisation (WHO); and grey literature, particularly concerning Malawi. In addition, one of the authors (DM) was part of a nine member external team established by the Government of Malawi and the UK Department for International Development to evaluate the country's antiretroviral therapy (ART) programme in September 2006. During the evaluation a number of health facilities were visited and informal interviews and discussions with service providers, managers and policy makers were conducted.

## Findings

### The twin human resource and HIV burden

The 2006 World Health Report (WHR) defined health workers as 'the people whose job it is to protect and improve the health of their communities' [1]. While recognising the important role of unpaid carers such as mothers and voluntary health workers, its analysis is restricted to people engaged in paid activities. Among those, two categories are identified: 'health service providers' who deliver services; and 'health management and support workers' who are not engaged in any direct provision of services. Table 1, reproduced from the WHR, summarises data on the availability of health workers by region and by the categories mentioned above. It suggests that regions with more health workers have proportionately more managerial and support workers. However, better data are required before any conclusions can be made about the number and relative availability of 'health management and support workers'.

**Table 1:** Global health workforce, by density

**WHO Region**	**Total health workforce**		**Health service providers**		**Health management and support workers**	
	
	**Number**	**Density ** **(per 1000 population)**	**Number**	**% of total health ** **workforce**	**Number**	**% of total health ** **workforce**
**Africa**	1 640 000	2.3	1 360 000	83	280 000	17
**Eastern Mediterranean**	2 100 000	4.0	1 580 000	75	520 000	25
**South-East Asia**	7 040 000	4.3	4 730 000	67	2 300 000	33
**Western Pacific**	10 070 000	5.8	7 810 000	78	2 260 000	23
**Europe**	16 630 000	18.9	11 540 000	69	5 090 000	31
**Americas**	21 740 000	24.8	12 460 000	57	9 280 000	43
**World**	59 220 000	9.3	39 470 000	67	19 750 000	33

Note: All data for latest available year. For countries where data on the number of health management and support workers were not available, estimates have been made based on regional averages for countries with complete data.

Data sources: World Health Organization. *Global Atlas of the Health Workforce *

**Figure 1:**
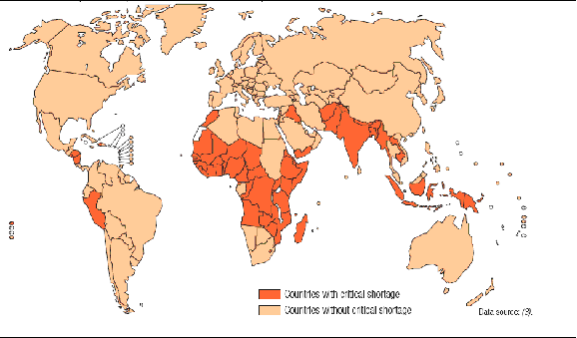
**Countries with a critical shortage of health service providers (doctors, nurses and midwives)**. (Source: World Health Report (2006), Working Together for Health, Geneva: WHO [1]).

The WHR also identified those countries with a 'critical shortage' of health workers (see Figure 1). Critical shortage was defined as having less than 2.28 doctors, nurses and midwives per 1000 population, a threshold derived from an analysis of workforce density associated with key public health outcomes by the Joint Learning Initiative [2] (see Table 1).

The WHR suggest that there are critical shortages of health workers in many countries. The absolute shortage is greatest in Asia, where there is a shortfall of 1.16 million doctors, nurses and midwives and perhaps 2.1 million of all types of health workers, dominated by the shortages in Bangladesh, India and Indonesia. The relative shortage is greatest in sub-Saharan Africa where an increase of 139% is required [1]. The countries with the lowest ratios of health workers per 1000 population are mainly in sub-Saharan Africa but some others such as Indonesia and Papua New Guinea also have densities below one half of the proposed critical shortage threshold.

The focus on doctors, nurses and midwives reflects the greater reliability of estimated numbers of these cadres. In practice, the contribution of other cadres such as pharmacists, laboratory technicians and 'non-physician clinicians', is just as critical. Indeed, 'non-physician clinicians' (often known as 'clinical officers' or 'medical assistants') have been trained in some countries to compensate for the lack of doctors and are active in 25 of 47 sub-Saharan African countries included in a recent study [3]. In nine countries there are more non-physician clinicians than physicians and they are reported to play prominent roles in primary health care and HIV/AIDS treatment in five of the worst affected sub-Saharan countries. However, in no country do they add more than 0.2 to the health worker per thousand population ratio, so they do not significantly alter the relative position of different countries from WHO's analyses.

Further analysis of data from WHO's global atlas of the health workforce identifies the countries in Table 2 as having ratios of doctors, nurses and midwives lower than 0.5 per 1000 population. All of these are in sub-Saharan Africa. Malawi has a slightly higher overall health worker density than these countries at 0.61 per thousand population, but a physician density level as low as the least well served of these countries (Niger) at 0.02 per thousand population [3] (see Table 2).

**Table 2:** Countries in the deepest human resource crisis according to their numbers of doctors, nurses and midwives: ratios per thousand population.

	**Physicians**	**Nurses**	**Midwives**	**TOTAL**
**Burundi**	0.03	0.19	0	0.22
**Ethiopia**	0.03	0.2	0.02	0.25
**Niger**	0.02	0.2	0.03	0.25
**Chad**	0.04	0.24	0.04	0.32
**Liberia**	0.03	0.17	0.13	0.33
**Mozambique**	0.03	0.21	0.12	0.36
**Senegal**	0.06	0.25	0.07	0.38
**United Republic of Tanzania**	0.02	0.3	0.07	0.39
**Togo**	0.04	0.33	0.05	0.42
**Rwanda**	0.05	0.42	0.01	0.48
**Central African Republic**	0.08	0.23	0.18	0.49

Source: Author's analysis of HRH global atlas, latest year available

The focus on nurses, doctors and midwives also runs the risk of neglecting the importance of 'health management and support workers'. Clinical workers require management and administrative systems to work if they are to be effective. And ART programmes require, in particular, effective drug procurement and supply systems, laboratory support and information management.

**Figure 2:**
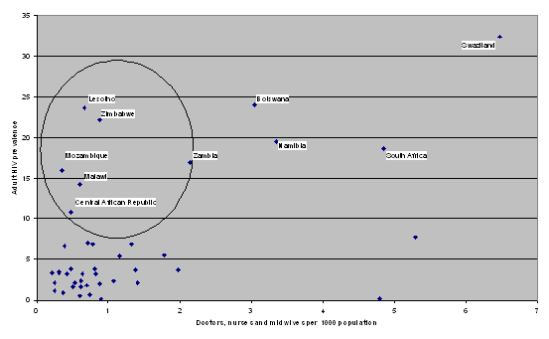
**Total numbers of doctors, nurses and midwives against adult HIV prevalence across African countries for which both statistics are available**. (Source: Authors' analysis based on HRH global atlas and UNAIDS data).

When countries with low HR levels are assessed in terms of HIV prevalence, all the non-African countries with a critical human resource shortage are found to have relatively low adult HIV prevalence rates. According to UNAIDS [4], adult HIV prevalence ranges from less than 0.1 to 1.6% in these countries except Haiti, where prevalence is 3.8%. The twin burden of HRH crisis and HIV/AIDS crisis is therefore an African phenomenon. Figure 2 plots total numbers of doctors, nurses and midwives against adult HIV prevalence across all African countries for which both statistics are available. It identifies 6 countries with an HRH crisis as defined by WHO and with adult HIV prevalence rates greater than 10%. These are Lesotho, Zimbabwe, Zambia, Mozambique, the Central African Republic and Malawi (see Figure 2).

Hirschhorn et al. [5] estimated that the additional health workforce required to deliver ART to 1000 patients amounted to 1–2 physicians, 2–7 nurses, <1 to 3 pharmacy staff and an unquantified number of counsellors and treatment supporters. On this basis, Mozambique, which needs to provide ART to about 200,000 patients, would require 200–400 doctors from its' total stock of 514, and 400–1400 nurses from its total stock of 3947.

**Figure 3:**
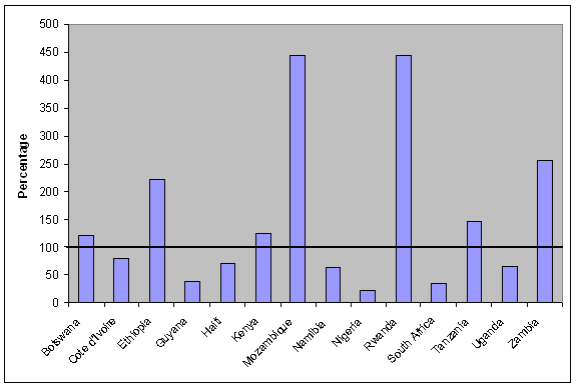
**Percentage of existing doctor workforce required for full coverage in 10 years**. (Source: Smith 2005 [6]).

Other estimates of the workforce required to scale up ART suggest even more stark results. Smith [6] calculated that seven out of fourteen countries included in his study would be unable to meet needs even if they used 100% of their current workforce. Figure 3 shows Smith's estimates of human resource requirements for full coverage of population with antiretroviral therapy. Only two of the 'twin burden' countries are considered in Smith's analysis – Mozambique and Zambia (Malawi was not included). Both are among the three countries whose current medical personnel situation appears least adequate for antiretroviral therapy expansion according to Smith. The third is Rwanda, one of the most human resource constrained countries (see Table 2), but with a relatively low estimated HIV prevalence rate. This estimate has recently fallen from a reported rate of 8.9% to 3.1% following the expansion of sentinel HIV surveillance to rural sites [7]; it is possible, but not clear, if Smith used the higher rate in his calculation (see Figure 3).

In part, these stark estimates reflect the clinical complexity and chronic nature of treating patients with AIDS. Even in the absence of antiretroviral therapy, HIV increases the needs for skilled human intervention in the health system, particularly due to the incidence of opportunistic infections. For example, one study in Rwanda estimated that 60% of hospital beds were occupied by AIDS patients being treated for opportunistic infections [8].

A comprehensive HIV/AIDS programme also includes a range of interventions unrelated to the treatment of people with AIDS such as HIV prevention strategies, including the comprehensive management of patients with other sexually transmitted infections, voluntary counselling and testing (VCT) services and the prevention of vertical transmission. All these interventions also require skilled health workers.

The HR requirements of ART programmes therefore have to be met within a severely limited pool of human resources. It is therefore unsurprising that the volume of additional funding and energy directed at HIV/AIDS programmes should threaten less well supported activities. Furthermore, the delivery of HIV/AIDS interventions through non-government organisations (NGOs) and private providers that are able to offer better pay and working conditions to health workers can lead to attrition from the public sector and other areas of health care [9,10].

### A case study of Malawi

#### Background

With an estimated GDP per head of US$646 in 2004, Malawi is one of the poorest countries in Africa [11]. Over half the 12 million population is food insecure and 65.3% were unable to meet their daily consumption needs in 1998 [12]. Life expectancy at birth is 39.8 years. HIV prevalence in Malawi was 14.1% (CI: 6.9 – 21.4) in 2005 [13]. The country is heavily dependent on aid which contributed 31.2% of Gross National Income in 2003, a higher proportion than most other countries in sub-Saharan Africa [14].

Malawi's health care indicators are poor [15-17]:

• Only 10% of health facilities were able in 2002 to deliver a basic minimum standard of care, with many being in poor condition, lacking an operational water source, electricity or a working telecommunications system.

• Full immunisation coverage has fallen from a rate of 81.8% in 1990 to 64.4% in 2004.

• The maternal mortality ratio is one of the highest in the world, standing at 984 per 100 000 live births in 2004.

• Only 46% of the population live within 5 km of a formal health facility and only 20% live within 25 km of a hospital

• 73% of households lacked an insecticide-treated bednet in 2004. According to one survey, more than 50% of malaria cases do not get treatment at health facilities.

There are however some notable achievements. Neonatal tetanus and polio have been eliminated through immunisation programmes and TB cure rates are over 70% [18]. And, as discussed later, there has been a great increase in the number of people living with AIDS receiving anti-retroviral therapy.

Malawi's health system is severely under-financed. In 2001, total health expenditure was US$ 12.4 per person [19]. At that time, the cost of delivering an 'essential health package' (EHP) of eleven cost-effective health services was estimated at $17.53 per capita, nearly 50% more than existing total health spending [20]. Furthermore, the cost estimate of this EHP was based on only 67% coverage for some services and did not include the costs of central level management and supervision, central hospital activity, or the provision of antiretroviral therapy.

According to WHO's National Health Accounts database, per capita total health expenditure in 2005 had risen to US$ 23. The government accounted for 24.3% of total health spending; donors/external funding for 51.5%; and private expenditure for 24.2% [21]. The organization of health care finances in Malawi has improved since 2005 as a consequence of a Sector Wide Approach (SWAp) which several donors, particularly DFID (UK), have agreed to support. Under the SWAp, a six-year programme of work was established, with the delivery of the EHP being at the core. However, not all external funding is channelled through the SWAp. USAID and PEPFAR are notable bilateral donors operating outside the SWAp framework.

In line with the focused international attention on HIV/AIDS, Malawi established a separate National AIDS Commission (NAC) to manage the significant amount of dedicated HIV/AIDS funding (including grants from the Global Fund) and to provide oversight over the country's HIV/AIDS plan. When first established, tension existed between the NAC and the Ministry of Health, partly because the NAC employed staff at higher salaries than the Ministry and because of the Ministry's loss of direct control over HIV/AIDS funding. According to a draft copy of Malawi's 2004/05 National Health Accounts, the Ministry of Health's share of public finance has decreased between 2002/03 and 2004/05 while that of the NAC increased (see Table 3).

**Table 3:** Share of public finance managed by different segments of the health system

**Budget management**	**Year**		
	
	**2002/03**	**2003/04**	**2004/05**
	**%**	**%**	**%**

Ministry of Health	60.2	49.5	51.6
National AIDS Commission	1.8	3.5	11.9
CHAM	4.2	2.9	4.2
Other NGOs	4.3	7.9	6.4
Donors	10.6	20	10.9
Other	18.9	16.2	15

(Source: Malawi 2004/05 National Health Accounts – draft copy (September 2006). Lilongwe: Malawi Ministry of Health)

Health services are provided by a multiplicity of providers. Of 'formal' health facilities, 60% are government-run; and 26% are mission facilities (mainly found in the rural areas). There is a small private-for-profit health sector (including three private hospitals) limited mainly to urban areas, as well as services provided by private companies for their employees. There is also a substantial traditional health sector. Nearly a quarter of deliveries are attended by a traditional birth attendant.

**Figure 4:**
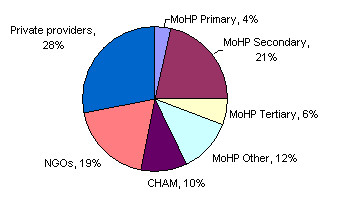
**Health expenditure in Malawi by provider sector, 1998/9 FY**. (Source: Government of Malawi, Ministry of Health and Population: Malawi National Health Accounts: a broader perspective of the Malawian Health Sector, 2001).

Mission facilities tend to operate independently of each other but within a loose association called the Christian Health Association of Malawi (CHAM). A formal agreement exists with the Ministry of Health whereby most of the CHAM workforce is paid from the government payroll. Other providers include islamic health facilities, NGOs, grocery stores, pharmacies and community-based distribution agents for contraception. The share of total health care expenditure in 1998/9 amongst different providers is shown in Figure 4. Since then, NGO health care provision has expanded, particularly NGOs providing HIV/AIDS services. There are also a number of clinical research projects, particularly related to HIV/AIDS in the health care system – these provide services to research subjects but also consume a significant number of the country's scarce skilled health workforce (see Figure 4).

In theory, health care providers in Malawi are organized according to a system of five 'zones' and 28 'health districts'. Each district is supposed to have an integrated health plan that incorporates the public sector, CHAM facilities and NGO providers. In practice, this does not always happen. Zonal offices which are supposed to provide support and supervision to district level services are relatively new and do not yet have the capacity to effectively support health districts. And in many districts, public, CHAM, NGO and private providers operate independently of each other.

Presently, local government assemblies provide a small amount of health services. However, there are plans to devolve primary health care provision to local assemblies, including the transfer of budgets and human resource employment responsibilities.

#### Human resources

As noted in the introduction, the total density of doctors and nurses (including midwives) in Malawi is 0.61 per thousand population, a shade higher than the threshold of 0.5 that defines the 10 worst served sub-Saharan African countries. As in other countries, the national average masks extreme inequities of provision within the country. In November 2004, 15 out of Malawi's 26 districts had less than 1.5 nurses per facility, and five had less than one. Only 13% of all health facilities had 24-hour midwifery coverage. Of 28 600 health worker posts in Ministry of Health (MoH) and CHAM facilities in 2005, about 38% were vacant [22]. Half of Malawi's doctors work at one of four central hospitals (although this partly due to deployment of newly qualified doctors to the central hospitals for the period of internship).

The HRH situation was described in April 2004 by the MoH as "dangerously close to collapse" and as a "major, persistent and deepening crisis" [23]. An independent review of a safe motherhood project concluded that in spite of "extensive staff training and support" to midwives, problems with staff retention would remain an important obstacle to increasing coverage of births by skilled attendants [24].

Three notable features of the health workforce in Malawi are the extensive use of clinical officers, medical assistants and about 4500 community-based health surveillance assistants (HSAs). Clinical officers receive four years of training and provide a range of medical services, including diagnosis and treatment, surgery and anaesthesia, and mending fractures. They form the cornerstone of hospital care in many rural areas. Medical assistants receive two years of training and mainly provide medical care in health centres and the outpatient departments of district hospitals. HSAs receive 10 weeks of training and are responsible for a variety of different tasks ranging from health promotion activities to TB defaulter tracing.

There are several reasons for Malawi's health worker crisis. One is its low resource base which has made it difficult for the government to adequately fund the training, employment and retention of health staff. Even after establishing a medical school in 1991, Malawi produced only 20 doctors per year until 2005. Although it produced about 40–60 registered nurses and 300–350 enrolled nurses annually in the early 2000s [25], this is small compared to an establishment of 8,963 public sector nurses (including CHAM) [23].

Another reason is HIV/AIDS. A 2002 study showed annual death rates of 2% among hospital health care workers [26]. Fear of exposure to HIV, particularly as shortages of gloves and other supplies hampers adherence to universal precautions, is also said to have contributed to staff leaving the sector [27]. Staff time is also lost to funeral attendance, care of sick family members and prolonged periods of illness. The increased workload caused by HIV/AIDS has also contributed to further demotivation, although according to local informants, the ability to treat patients with antiretroviral therapy is said to have improved staff morale.

Staffing problems are more acute in the public sector. Whereas 20 years ago, public sector health worker salaries were considered attractive, wages for civil servants in Malawi have not kept up with rising consumer prices [28]. Job opportunities in the better-paid private sector (including NGOs and research institutions) and abroad, particularly in the UK, have been another potent 'pull factor'.

Other reasons staff leave the public sector include poor working conditions; infrequent supervision and support; the lack of essential drugs, supplies and equipment; limited career progression opportunities; unequal access to training; an unclear deployment policy; and poor housing [29]. A study conducted in early 2006 identified up to 740 'inactive' professional health workers, including 469 nurses and 164 Clinical Officers, who had either resigned or retired from the health sector [30].

#### The Emergency Human Resources Programme

Malawi has implemented a variety of initiatives to solve its health worker shortages over the years. However, it was only after Peter Piot, Executive Director of UNAIDS, and Suma Chakrabarti, Permanent Secretary of the UK Department for International Development (DfID), visited Malawi in 2004 and witnessed first hand the hopeless staffing situation of many facilities that a substantial human resources plan was pulled together. The result was a shift from piecemeal donor support to a comprehensive six-year "Emergency Human Resources Programme" (EHRP).

Costed at US$272 million, with major funding from DFID and some from the Global Fund, the EHRP aims to raise Malawi's staffing levels (see Table 4) to a point where it could deliver the EHP (the planned targets do not therefore cater for the additional staff needed to provide antiretroviral therapy services). Although the EHRP would significantly boost staffing levels, the targets still fall short of the WHO-recommended minimum (on a rough estimate the EHRP would increase the total doctor and nurse density to 1.51 compared to the 2.28 threshold used by the 2006 WHR to define a 'critical shortage') (Table 4).

**Table 4:** Selected EHRP staffing targets (F/Y 2005–2006 Stock Indicator)

**Category**	**Combined Ministry of Health & CHAM**		
	
	**EHRP Target**	**Filled**	**Vacancy Rate (%)**
Physician/Specialist	433	162	63%
Nurse (all categories)	8,440	3,416	60%
Clinical Officer	1,405	1,033	26%
Medical Assistant	1,500	491	67%
Radiography/Technician	270	58	79%
Pharmacy/Technician	269	134	50%
Medical Laboratory Technician	507	182	64%
Environmental Health Officer	1,662	223	87%
Dental Technician/Therapist	470	138	71%
Physiotherapy	168	22	87%
Medical Engineering	60	24	60%
Health Surveillance Assistant	11,000	4,664	58%

(Source: Government of Malawi, Ministry of Health (July 2006). Strategic human resources for health framework for the health sector)

The EHRP takes a five-pronged approach:

• Improving incentives for recruitment and retention of public sector and CHAM staff through a 52% salary top-up for 11 professional and technical cadres, coupled with a major initiative to recruit and re-engage qualified Malawian staff.

• Expanding domestic training capacity, including doubling the number of nurses and tripling the number of doctors in training.

• Using international volunteer doctors and nurse tutors as a short-term measure to fill critical posts while Malawians are being trained.

• Providing technical assistance to bolster Ministry of Health (MoH) capacity in human resources planning, management and development.

• Establishing robust human resources monitoring and evaluation capacity.

In addition, the programme explicitly recognises the importance of improving policies on postings and promotions; training and career development; and incentives for deploying staff to underserved areas (which includes a major effort to improve staff housing). Technical assistance to the MoH in the form of human resources experts was therefore arranged. The government has also introduced a period of compulsory public health service for enrolled nurses trained at public expense.

Contrary to initial fears, other public servants did not protest at the improved pay for health workers partly because of the careful way in which the government and others had made the case for higher pay for health workers [31]. However, any further improvements to the pay and working conditions of health workers are likely to be resisted without improvements for other civil servants.

Since its implementation, anecdotal reports indicate that the salary rise had helped stem the flow of staff, particularly nurses, out of the public sector [31]. In addition, by the last quarter of 2005, 591 'inactive' staff had been recruited and more than 1,100 staff had been promoted (mostly nurses whose promotions had been blocked by civil service rules following a change to the nursing curriculum).

The number of health professionals trained annually increased from 400/year in 2004 to over 1000/year in 2006. The College of Medicine increased its first-year Medical Doctor intake for 2005 to 60 students [22]. By mid-2006, health-training institutions were running at full capacity, albeit with a need to improve tutor: student ratios. To further increase the output of nurse training institutions, proposals exist to reduce the length of time required for basic nurse training from four to three years (longer than in most other African countries). In 2006, 51 expatriate doctors and 15 nurse tutors were scheduled to be in post [31].

However, a recent evaluation of the EHRP in 2006 concluded that there were still difficulties in attracting tutors, doctors and nurses and that the EHP would not succeed if "radical action is not taken to dramatically improve retention rates", particularly in rural areas [32]. Another illustration of on-going problems was the observation that although expatriate doctors had been recruited successfully through the UN Volunteer Programme, in 2004/05 and 2005/06 less than eight medical graduates had joined the MoH whilst several other junior doctors had resigned [33].

According to Medecins Sans Frontieres (MSF), in Chiradzulu district, there were 50 nurses working at the district hospital in 2006; that number had dropped to 28 by 2007 [34]. MSF also noted the experience of retired nurses who had been attracted to return to the workforce having trouble getting contracts and payment due to administrative delays.

One problem was that the promised 52% salary top-up was not translated into a 52% increase in take-home pay because of changes to the tax and allowance structure of public sector health workers. Furthermore, in spite of the salary top-ups, non-government employers still offer much better rates of pay, particularly for scarce health worker cadres such as doctors, laboratory technicians and pharmacists.

The fragmented and competitive provider market, coupled with the pressure on funders and policy makers to achieve ambitious coverage targets, has caused the labour market to become extremely uneven. Scarce skills appear to be concentrated in urban areas and in NGO/research projects that are able to offer higher remuneration. According to MSF, external financing is also associated with workshops and training programmes which public health workers are paid with per diems and stipends to attend. A five-day training workshop can increase a nurse's basic monthly salary by 25–40% [34]. Although training workshops are necessary, the competition for stipends can disrupt service delivery and increase absence from facilities.

#### HIV/AIDS and the provision of antiretroviral therapy

In spite of its significant health systems constraints, Malawi has made exceptional progress in expanding access to ART. At the end of 2006, there were about 60 000 people on treatment in the country, with plans to expand coverage to 245 000 people by 2010.

This progress is argued to have been achieved because of several factors [35]:

 A strong rights-based international advocacy movement

 Earmarked funding for antiretroviral therapy services from a range of donors.

 Support from international NGOs and research organisations to deliver ART services

 Strong technical leadership and management within the Ministry of Health

 A vertical management and delivery system which has included:

◦ dedicated ART training programmes for various cadres of health workers (see below)

◦ A stand-alone system for financing, procuring and distributing antiretroviral therapy drugs. This involves drugs procured by UNICEF from India being flown to Copenhagen where they are individually packed for each ART clinic, and then flown to Malawi where they are couriered to each ART clinic.

◦ A stand-alone information system to enable high-quality monitoring and evaluation

◦ Quarterly supervision and support visits to all ART clinics

 A 'low-resource approach' which includes using a single first-line and second-line regimen for all patients and providers; using clinical staging to determine eligibility for treatment (not CD4 counts); using fixed-dose combination tablets; and using clinical signs only to monitor treatment response.

Under the direction of the HIV/AIDS unit within the MoH, an agreement has been reached that ART providers will be supplied with government-procured drugs, whether in the public or private sector, provided they attend a 5-day training course and formal assessment. In the private sector, in addition to paying private consultation fees, patients pay a fee of MK 500 (at time of writing, US$ 1 = MK 140) per month for the medicines, of which MK 200 is retained by the private provider and MK 300 is paid into a revolving fund managed by the Malawi Business Coalition Against HIV/AIDS which is then remitted to the National AIDS Council. The cost of ART on the government procurement scheme is approximately MK 1820 per month, although this excludes the costs of supply and distribution logistics [36].

Before new ART sites are established, those responsible for establishing the sites and providing care must also spend two weeks attached to one of the specialist HIV centres within Malawi after completing the 5-day training course. Through the provision of subsidised medicines and using this model of structured training, the government has been able to harness the private sector to support the national ART programme.

Human resource plans to further expand ART coverage involve four main strategies [35]:

1) minimizing the health worker: patient ratio by changing the requirement for all patients to be seen by a clinician when they come for repeat prescriptions.

2) 'task-shifting' to enable nurses to diagnose and prescribe ART and 'lower' cadres of health workers (in particular HSAs) to dispense ART. Plans exist to overcome legal and professional restrictions on prescribing and dispensing, and to train and equip HSAs with the competencies to provide ART drugs; keep accurate patient records; and question carers and patients so that an appropriate treatment regime and referral pattern can be established. [However, it should be noted that this is being resisted by the Pharmacy, Medicines and Poisons Board]

3) increasing the number of health workers involved in the ART programme by including volunteers/unpaid workers.

4) decentralizing management and supervision to zonal and district health management structures.

In Thyolo district, MSF has been working with the government to provide more than 10 000 people with ART by the end of 2007. One of its strategies has been to support 600 volunteer community home-based caregivers to assist community nurses with the management of common HIV-related conditions, support people on ART, and trace defaulters [37]. Nurses are also being used to manage 'stable patients' (defined as non-pregnant adults who have been on first-line treatment for at least one year with no complications or adherence problems).

However, as Malawi contemplates the further expansion of antiretroviral therapy (whilst sustaining its current gains) within the context of limited resources and many shortfalls in the provision of other essential health services, there are concerns that these other services could be harmed. In addition, the intention to decentralize new responsibilities to zonal and district offices might compromise this management capacity which is already struggling to oversee and support other health programmes.

The external evaluation of Malawi's ART programme in 2006 noted concerns that antiretroviral services resembled 'islands of excellence in a sea of problems' [35]. While the ART programme's achievements were impressive, other services (including the prevention of vertical transmission) showed signs of stagnation. One contrast was the excellent supply of ART drugs compared with the abysmal supply of other essential health commodities; another was the plans to up-scale paediatric ART when it was clear that the country's programme to reduce vertical transmission had stalled. It was also noted that the ART programme's focus on individual treatment had under-emphasised the potential for treatment services to act as an engine for HIV prevention.

However, the ART programme could also impact positively on the health system by, for example, helping keep HIV-positive health workers healthy and preventing facilities from being overwhelmed by the needs of people dying from AIDS. In addition, the political and civic energy and additional resources directed at the scale up of ART provides an opportunity to strengthen health systems. For example, the impetus to reduce vertical HIV transmission can be harnessed to improve the quality of ante-natal and obstetric care as a whole.

The recent introduction of eight paediatricians from the United States to help increase coverage of paediatric ART is another example of how the current international focus on AIDS treatment could be harnessed to strengthen the health system as a whole. As well as increasing paediatric ART coverage, these physicians could be deployed to support the improvement and expansion of other child health services.

## Conclusion

Malawi is one of a few countries in extreme crisis in relation to both human resources and HIV. Despite this, it is making remarkable progress both in tackling the causes of human resource problems and in providing antiretroviral therapy. The case highlights how both synergies and conflicts between the two strategies have been realised in this context out of the many possible configurations that could be theoretically predicted.

Among the evident synergies is the contribution of the EHRP to increasing the availability of staff for the expanding ART programme both in total and in underserved areas. This is consistent with the growing acknowledgement that the basic human infrastructure of health systems in SSA must be strengthened if global ART coverage targets are to be met.

However, while Malawi has been fortunate enough to receive donor support for its EHRP, it is clear that more needs to be done. Donors and the government still need to secure additional resources to increase staff recruitment and training, and to improve retention if WHO's minimum HRH standards are to be met. In addition greater attention will need to be paid to the dynamics of the domestic labour market and the disparities between private and public sector remuneration if Malawi is to ensure a more rational and needs-based deployment of health workers across the country.

The impact of external project funding on workforce balance has been suggested to lead to the need for health workforce impact assessments as part of project appraisal: 'It could be envisaged that at country level, public and private health services, NGOs and international agencies that would like to start up a new programme or activity would have to demonstrate the impact of their plan on the current health workforce to the Ministry of Health. Similarly, organizations applying for funding at international donor agencies would be asked the same' [38].

The adoption of a low-resource model of ART provision, underscored by task shifting and the use of clinical officers and nurses to diagnose and treat AIDS patients is also consistent with protecting staff availability to also meet other demands on the health system. Moreover, the effective delivery of ART should reduce demands on the health care system for the treatment of opportunistic infections and end-of-life care, although this may only be a short term impact. As the ART programme matures, the number of treatment failures to first-line regimens will grow and unless there is the capacity to fund and supervise the use of second-line treatment, the health care system could see a 'rebound effect' of returning AIDS patients.

Another notable achievement of the Malawi ART programme has been its ability to incorporate private sector providers into the national ART programme. This has been achieved through a quid pro quo arrangement whereby private sector providers agree to adopt national treatment and monitoring policies in exchange for medicines that are mainly paid for by the public purse.

The potential for synergy in the strengthening of drug supply systems has not yet been realised. The procurement and supply system for ARVs described earlier operates in isolation from the procurement and supply system for other medicines and commodities, which includes a number of other stand-alone, vertical systems.

Among the evident conflicts are the demands placed on service delivery by additional in-service training activities and the conflicts between dedicating resources to ensuring the excellence of the antiretroviral therapy programme, and ensuring excellence in prevention – especially of mother-to-child transmission which could reduce the demands for a paediatric antiretroviral therapy programme. There is perhaps a more important tension between the patient-centred focus required for ART, and the community centred approach required for prevention programmes that target the broader determinants of HIV infection and other health problems. This may prove more difficult to resolve.

Planning for the rapid scale-up of antiretroviral therapy whilst simultaneously strengthening health systems and delivering the broader EHP will require prioritizing amongst the different health care needs and careful consideration of coverage targets and timeframes set by the antiretroviral therapy programme because it takes time (not just resources) to develop positive synergies between antiretroviral therapy scale-up and health systems strengthening. It will also require the right balance between the use of 'dedicated' antiretroviral therapy cadres of health workers and an antiretroviral therapy programme operated through 'generalist' health workers capable of providing comprehensive care. The former may result in faster and more effective antiretroviral therapy coverage, but the latter may be more sustainable and be less harmful to other health care services.

A greater emphasis on sustainability and health systems strengthening may compromise the speed of up-scaling in the short-term but could derive greater benefits in the long-term by, for example, ensuring a high level of treatment adherence, keeping patients on first line treatment for longer and deferring the use of more expensive second-line treatments.

## Abbreviations

ART: antiretroviral therapy; CHAM: Christian Hospital Association of Malawi; EHP: essential health package; EHRP:  Emergency Human Resources Programme; GDP: gross domestic product; HSA: health surveillance assistants; MSF: Medecins Sans Frontieres; NAC: National AIDS Commission; NGO: non-government organisations; VCT: voluntary counselling and testing; WHO: World Health Organisation; WHR: World Health Report; SWAp: Sector Wide Approach.

## Competing interests

The authors declare that they have no competing interests.

## Authors' contributions

DM and BM drafted the paper on the basis of two separate papers they had individually authored. VM made significant contributions to the original draft with particular reference to the Malawi case study.
